# Male partners’ involvement in birth preparedness and complication readiness plan in Dale district, Sidama regional state, Ethiopia, 2021

**DOI:** 10.1186/s12905-024-02941-3

**Published:** 2024-03-13

**Authors:** Daniel Yehualashet, Hirut Gemeda, Berhan Tsegaye Negash

**Affiliations:** 1 Department of Midwifery,Collage of Medicine and Health Science, Dilla University, Dilla, SNNPR Ethiopia; 2 Department of Midwifery,Collage of Medicine and Health science , Hawassa University, Hawassa, Ethiopia

**Keywords:** Male partner, Involvement, Birth preparedness, Complication readiness

## Abstract

**Background:**

Male partners’ involvement in birth preparedness and complication readiness plans is a key strategy to improve maternal and child health. It assists an expectant mother to make timely decisions in receiving care where service is inaccessible. Despite its significance, information is scarce about male partner involvement in birth preparedness and complication readiness plan in the study setting.

**Objective:**

To assess prevalence and factors associated with male partners’ involvement in birth preparedness and complication readiness plan in Dale district Sidama, Ethiopia in 2021.

**Methods:**

A community-based survey was done from November to December, in 2021. Data were collected using a structured, pre-tested and interview administered questionnaire. A multi-stage cluster sampling was applied to recruit 634 samples. Logistic regression analysis was performed to identify factors associated with male partner involvement in birth preparedness and complication readiness plan. Adjusted odds ratios (AORs) and 95% confidence intervals (95% CI) of associated factors were estimated by stepwise backward likelihood ratio method.

**Results:**

622 out of the 634 study participants completed the interview, yielding a 98.1% response rate. Prevalence of male partners’ involvement in birth preparedness and complication readiness plan was 47.6% (95%CI: 44.9%, 48.9%). After adjusting the cofounding variables, factors like accompanying wives with their partners during their antenatal care visits (AOR = 2.3, 95%CI 1.5, 3.5), male partners whose wives had a history of caesarean birth (AOR = 2.1, 95%CI 1.1, 3.8), knowledge of male partners on birth preparedness and complication readiness plan (AOR = 3.5, 95%CI:3.1,6.6), presence of obstetric complications(AOR = 5.1,95%CI:4.3,11.2),primi-gravida (AOR = 2.7,95%CI:1.6,4.7), and male partners’ knowledge of obstetrics complications (AOR = 3.5,95%CI,2.2,5.7) were significantly associated with male partners’ involvement in birth preparedness and complication readiness plan.

**Conclusion:**

This study indicates that prevalence of male partners’ involvement in birth preparedness and complication readiness was low. Therefore, awareness creation should be strengthened on male involvement on birth preparedness and complication readiness plan.

## Background

Today, there are 830 maternal fatalities caused by avoidable complications during childbirth every day. The majority (99%) of these fatalities occur in nations in sub-Saharan Africa [1]. In industrialized countries, the Maternal Mortality Ratio (MMR) in 2015 was 12 deaths per 100,000 live births. However, in sub-Saharan Africa, the MMR was projected to 546 [3]. Therefore, pregnancy, childbirth and postpartum periods are still the main concerns of women who live in sub-Saharan countries [2, 3]. In Ethiopia, MMR was 420 per 100,000 live births in 2011 [4].

Birth preparedness and complication readiness plan (BPCRP) is a comprehensive package aimed at promoting timely access to skilled care, active preparation and decision making for delivery by pregnant women and their families [4]. It is therefore a strategy to promote the timely use of skilled maternal care and preparing for childbirth reduces delays in obtaining this care [5]. It measures the level of readiness for birth and complication not only expectant mothers, but also the extent of participation of their partners, families and communities [4, 6]. It consists of two separate but related concepts: birth preparedness includes items like identification of a skilled birth attendant, place of delivery, and materials needed during childbirth [7]. On the other hand, complication readiness plan include awareness on the key danger signs and preparation of ,money, transport and potential blood donor and a decision-maker personnel [8].

Male involvement in birth preparation and the readiness plan for complications is crucial in lowering maternal mortality ratio [9]. Particularly, men are responsible for deciding whether to use maternal health services for their wives in Africa [2]. In Africa, delays in finding money and potential blood donors in times of need, transportation, and getting to the right referral hospital continue to be significant challenges [10, 11]. For example, most of decision regarding financial resources, family size, and use of health services are made by men in most patriarchal societies of Ethiopia [3]. Couples usually worry about improving pregnancy outcomes but it tends to be seen as women responsibility, particularly in most rural settings [11]. Husbands urge their wives to access emergency obstetric care [12], encourage their wives to seek services early if they are involved in birth preparedness and complication readiness plan [7].

Based on World Health Organization (WHO) description male involvement in health service utilization has an indirect positive impact on other health outcomes [9]. Evidence suggests that men participation in maternal health service increase proportion of institutional deliveries [13, 14] and postnatal care visits [14, 15]. For instance, men in health education for pregnancy danger sign is the pivotal strategy for sustainable decision making process for health service utilization [7]. However, previous study report indicated that only one from five pregnant women (21%) attend prenatal care clinics with their husbands in Ethiopia [16].

On the contrary, policy makers have paid little attention about husband engagement in birth preparedness and complication readiness plan in Ethiopia [10, 16, 17]. Moreover, information is scarce on male involvement in birth preparedness and complication readiness plan in the study area [14, 15, 18]. Therefore, this study aimed to assess prevalence and factors associated with male partner’s involvement in birth preparedness and complication readiness in Dale district Sidama, Ethiopia, in 2021.

## Methods

### Study area, design and period

A community-based survey was done among partners of women who had children of younger than 12 months in Dale district, Sidama, Ethiopia, from November to December 2021.

The study took place in Dale district, which is situated in Sidama region, 328 km from Addis Ababa, Ethiopia’s capital. The district has 3 urban and 36 rural Kebeles. Agriculture is the main livelihood source for the population. The total population of the district was estimated at 272,212. There were 1452 women who gave birth in the previous 12 months in the study setting. There are 1 hospital, 10 health centers, 36 health posts, and 3 private health institutions that provide preventive and curative services in the community. The potential health service coverage of the district was estimated as 70% in 2017.

### Population

All husbands whose wives delivered in the last 12 months in the selected Kebeles of Dale district and had resided for more than 6 months were included in this study. However, study participants who were critically ill during data collection period and living with their wives were excluded from the study.

### Sample size determination and sampling procedure

The sample size was determined using a single population proportion formula. The sample size was estimated based on the following key assumptions: prevalence of male partners’ involvement in BPCRP (*P* = 50.8%) [17], 5% margin of error, and 95% confidence level.

The standard Cochran formula was applied to calculate initial sample size in this study (n = pq/d^2^). After plugging the values mentioned above into formula, an initial sample size of 384 was computed. Then, we have added 10% contingency for the non-response rate and 1.5 design effects as compensation making the overall sample size of 634.

As the sample sizes for second objective were lower than sample size calculated for prevalence, we have taken sample size for prevalence in this study. A two stage cluster sampling strategy was applied in this study. Ten (30%) rural Kebeles were selected by lottery method. First, Simple random sampling method was used to select participating Kebeles. Finally, husbands were reached through their spouses. Study participants were selected based on the identification of their wives from family folders of health extension workers in their respective Kebeles.

Complete and updated lists of women with their household were utilized as a sampling frame in this study. Unique identifier number was given for each household. Finally, from the list, women who gave birth in the last 12 months were selected randomly using computer generated technique and their husbands interviewed in a private place around their home. Those husbands who were not available at first visit were revisited for the second times and those who were not available were revisited for the third times.

### Data collection tools and procedures

Data was gathered using a pretested, structured, standard and interview administered questionnaire. The questionnaire was adapted from Johns Hopkins Program for International Education in Gynecology and Obstetrics (JHPIEGO) guideline [19]. It consists of socio-demographic charactestics, reproductive health charactestics and individual characteristics like knowledge of obstetric danger signs and knowledge of birth preparedness and complication readiness plan. The questionnaire was prepared first in English language, and then translated to Amharic and Sidaamu Afoo (local language). Then, it was back translated to keep it consistency. Data was collected by 8 BSc midwives and supervised by 2 postgraduate clinical midwives. Data collectors were fluent for Sidaamu Afoo and Amharic languages. For study participants who were not found at home during the first visit, the data collectors revisited 2–3 times before reporting them ‘non-respondents’.

### Operational definitions

#### **Male partners’ involvement in birth preparedness and complication readiness plans**

is defined as being available physically with their wives in a health facility to give the physical, emotional, and economical support that enables them to access the routine antenatal care, delivery care, and postnatal care services from skilled health workers [1].

#### **Adequate involvement in birth preparedness and complication readiness plan**

 Access who practiced at least four components from the seven parameters of birth preparedness and complication readiness plan; otherwise, inadequate involvement in birth preparedness and complication readiness plan. These include the following components: being personally accompanied, identifying skilled care provider, selecting the preferred birth place, preparing clothes and other materials, identifying means of transportation, arranging household support, and making blood donor made ready [20].

#### **Kebele**

The lowest government administrative hierarchy that exists next to a district.

#### **Knowledge of birth preparedness and complication readiness plan**

is a strategy to encourage husbands to be informed of the danger signs of obstetric complications and emergencies, choose a preferred birth place and attendant at birth, arrange for transport to the skilled care site in case of an emergence, save or arrange alternative funds for costs of emergency care, and accompany her to emergency care. Identifying a blood donor, preparing clean clothes for the mother /mother’s baby and arrange a source of household support to provide temporary family care during her absence [21].

#### **Knowledge of danger signs during pregnancy**

A husband is considered knowledgeable if he spontaneously mentioned all the three key danger sign of pregnancy, such as vaginal bleeding, blurred visions and swollen hands/face otherwise not knowledgeable [21].

#### **Knowledge of danger sign during labor**

A husband is considered ‘knowledgeable’ if he spontaneously mentioned four key danger signs of labour: severe vaginal bleeding, convulsions, prolonged labour and retained placenta, otherwise, not knowledgeable [21].

#### **Knowledge of danger sign during postpartum**

A husband is considered ‘knowledgeable ‘ if he can mentioned all three key danger signs in postpartum period: as severe vaginal bleeding, foul smelling and high fever otherwise not knowledgeable [21].

#### **Male partner**

is defined as the biological or non-biological father of woman’s child who is considered a ‘partner’ by the woman at the time of data collection.

#### **Kebele**

is the smallest administrative unit in Ethiopia, which consisting of 1000 households.

### Study variables

The dependent variable of this study was male partners’ involvement in birth preparedness and complication readiness plans. It is the binary outcome variable. It is the proxy variable, which consists of seven parameters. Each parameter was scored as a dichotomous variable and coded ‘1’ if the parameter is actually implemented by a women partner; otherwise, ‘0’. Those partners who scored 4 parameters and above were recoded as ‘1’, otherwise, ‘0’.

Independent variables measured in this study consist of socio-demographic variables, reproductive health variables, individual variables, and health facilities-related characteristics. The socio-demographic variables include the age of couples, educational status, occupation, household income, and religion. Besides, reproductive health variables consisted of place of delivery, decision-maker on place of delivery, source of information, heard about obstetrics danger signs and problems prevented from going to health facilities with wives. Lastly, individual variables included partners’ knowledge of birth preparedness, and complication readiness plan, and obstetrics danger signs. Health facility-related factors include the presence of health facilities in households, and the time to reach health facilities.

### Data quality control

The quality of the data was assured through the proper design and pre-testing of the questionnaire on 32 participants on Wondogent district prior to data collection. Data collectors were trained for 2 days on different issues: objective, relevance, and benefits of the study; confidentiality of information; respondent’s right; informed consent; and technique of interview. Morning meetings, led by the principal investigator, were held with all field workers throughout the data collection periods and continuous concrete feedback was provided on the spot. Filled questionnaires were checked daily for completeness, legibility, and consistency.

### Data analysis

Data were coded, cleaned, and entered using Epi-data version 4.6 and exported to SPSS version 25 for analysis. Descriptive statistics were summarized using frequencies, mean, standard deviation, and 95% confidence intervals. Data are presented using charts and tables. The chi-square test was checked for the presence of association using contingency tables for categorical variables. We initially retained all variables that were significant at P < 0.25 in binary logistic regression analysis. We then used the enter method to select variables in the multivariate logistic regression model. This was repeated until all variables in the model had a P-value of < 0.05. Multicollinearity was checked to be absent using Variance Inflation Factor (VIF) and tolerance levels (TLs), with multicollinearity defined as VIFs > 2.5 and TLs < 0.40 [22]. Hosmer Lemeshow Goodness-of-fit was checked to test the model fitness. Crude and Adjusted Odds Ratios (AOR) with their 95% Confidence Interval (CI) were calculated to determine the strength and presence of association.

## Results

### Socio demographic characteristics

Among the total of 634 study participants, only 622 study subjects were interviewed completely making a response rate of 98.1%.

Table 1 of this study highlights the summary of socio-demographic characteristics of the study participants. The mean age of the study participants was 29 **±** 6 years. Most (83.5%) of the study participants were married, and nearly one third (32.7%) of the study participants completed high school education. Regarding to religious affiliations, majorities (61.1%) of the study participants were Protestants followed by Ethiopian Orthodox Christian (21.1%) and Muslim (13.5%).Concerning occupation, most (58.5%) of the study participants were farmers. Besides, more than one third (31.7%) of the study subjects do private business (See Table 1).

**Table 1:** Distribution of male partners’ and wives socio demographic and economic characteristics in Dale district, Sidama Regional State, Ethiopia, 2021(*n* = 622)

Variables	Characteristics	Frequency	Percentage
Age of husband (*n* = 622)	< 29 30–34 35–39 > 40	207 136 125 154	33.3 21.9 20.1 24.8
Age of wives(*n* = 622)	< 24 25–29 30–34 > 34	152 190 133 147	24.4 30.5 21.4 23.6
Religion(*n* = 622)	Protestant Orthodox Muslim Catholic Wakefata	380 131 84 15 12	61.1 21.1 13.5 2.6 1.7
Husband educational status(*n* = 622)	No formal education Primary (1–8) Secondary (9–12) Higher(+ 12)	120 246 178 78	19.3 39.5 28.6 12.5
Wives educational status (*n* = 622)	No formal education Primary (1–8) Secondary (9–12) Higher(+ 12)	42 337 147 96	6.8 54.2 15.4 23.6
Husband occupation (*n* = 622)	Farmer Private/own work Employee Student	364 197 43 18	58.5 31.7 6.9 2.9
Wives occupation (*n* = 622)	Household Private/own work Employee Other*	375 67 92 88	60.3 10.8 14.8 14.1
Income status (*n* = 622)	< 500 500–1000 > 1000	72 306 244	11.6 49.2 39.2
Income earner (*n* = 622)	Husband only Wife only Both Neither	119 73 318 18	19.1 11.7 51.1 18.0

Other**= Student + daily laborer

### Reproductive characteristics

Table 2 of this study displays the study participants’ individual characteristics. One-third (31.5%) of the study participants accompanied their spouses during antenatal care visits. Most spouses of husbands 562(90.4%) delivered their child through spontaneously vaginal delivery. One out of five (19.8%) wives of husbands has already encountered obstetric complications before data collection. Vaginal bleeding 60 (9.6%), perianal and tear 15(2.6%) were the most commonly mentioned complications. Two-hundred-seventy eight (44.7%) wives of males have delivered their previous child at home. One-fifth (19.3%) of wives of husbands preferred their delivery place. Two hundred thirty 230(37%) study participants have heard information about obstetrics danger signs during pregnancy, childbirth and postpartum. However, only 289(46.5%) wives heard from health extension workers (See Table 2).

**Table 2:** Spouses’ reproductive characteristics of male partners in Dale, Sidama, South Ethiopia (*n* = 622)

Variables	Categories	Frequency	Percent
Accompanied wife for ANC	Yes No	196 426	31.5 68.5
Place of delivery of last recent child	Home Health facility	278 344	44.7 55.3
Mode of last delivery	By SVD By C/S	562 60	90.4 9.6
Who decided place of birth	Husband only Wife only Both together Other *	125 120 324 53	20.1 19.3 52.1 3.5
Obstetric complication in prevous pregnancy	Yes No	123 499	19.8 80.2
Problem prevented from going to health facility with wife	Yes No	442 180	71.1 28.9
Pregnant woman is susceptible for complications	Yes No	435 187	69.9 30.1
Heard about obstetric danger signs, birth prepardness	Yes No	230 392	37.0 63.0
Source of information	Health extension Radio Television Discussion with wives	289 158 93 82	46.5 25.4 15.0 13.2

*Key* SVD-Spontaneous Vaginal Delivery, ANC-Ante-Natal Care, C/S-Caesarean Section, *-friends and health providers

### Health service characteristics

Table 3 of this study portrays the health service factors associated with male partners of women who had children younger than 12 months. Most (83.8%) of the study participants mentioned the presence of health facilities in their surroundings. However, more than a quarter of them traveled to the health facility. Nearly half (48.2%) of the study participants obtained information about birth preparedness and complication readiness plan from community health workers. Almost half (49%) of male partners’ wives had less than four antenatal care visits. Majorities (95.8%) of the study participants were not influenced by their wives (**see** Table 3).

**Table 3:** Health service related charactestics of the study participants in this study (*n* = 622)

Variable	Characteristics	Frequency	Percentage
Presence of health facility in the district	Yes No	521 101	83.8 16.2
Time to reach health facility	Less than 30 min. From 30 min to 1 h From 1 to 2 h. More than 2 h.	145 213 157 107	23.3 34.2 25.2 17.2
Source of information about BPCRP	Community health worker Media Friends and family	300 117 205	48.2 18.8 33
Number of ANC session of wives	Less than 4 4 and above	305 317	49 51
Presence of spousal influence to engage during maternity care	No Yes	596 26	95.8 4.2
Did husbands have previous experience in participation of maternal health service?	No Yes	485 137	78 22

*Key* ANC-Antenatal Care, BPCRP-Birth Preparedness and Complication Readiness Plan

### Individual health characteristics

The knowledge of study participants about obstetric danger signs was estimated to be 447 (71.9%), 436 (70.1%), and 423 (68.0%) during pregnancy, delivery, and the postpartum period, respectively. From the total study participants, 202 (40.5%), 109 (21.3%), and 136 (30.1%) mentioned vaginal bleeding, blurring of vision, and swelling of hands or faces, respectively, as key danger signs during gestation. At delivery, severe vaginal bleeding was mentioned by 419 (67.4%) of the study participants. Furthermore, convulsions were described by 322 (51.8%) study participants, prolonged labor lasting more than 12 h was explained by 383 (61.6%) study participants, and placentas were not delivered for more than 30 min by 376 (60.5%) study participants. During the postpartum period, the most commonly mentioned danger sign was vaginal bleeding (96.1%), followed by malodorous vaginal discharge (53.4%), and lastly, severe fever (50.5%) (**See** Table 4).

**Table 4:** Knowledge of study participants on pregnancy danger sign (*n* = 622)

Variables	Frequency	Percent
**During pregnancy**		
Vaginal bleeding	409	65.8
Swelling of g\hands/face	319	51.3
Blurring of vision	259	41.6
**During labor/delivery**		
Vaginal bleeding	419	67.4
Convulsion	322	51.8
Labor lasting > 12 h	383	61.6
Placenta not delivered 30 min after baby	376	60.5
**During postpartum**		
Vaginal bleeding	430	69.1
Malodourous vaginal discharge	332	53.4
High fever	314	50.5

### Male partner’s participants in birth preparedness and complication readiness plan

Male partner who practices at least four of the seven components of birth preparedness and complication readiness is categorized as knowledgeable. Regarding knowledge of child birth preparedness and complication readiness plan, 318 (51.1%) study participants were found to be knowledgeable.

**Fig. 1:**
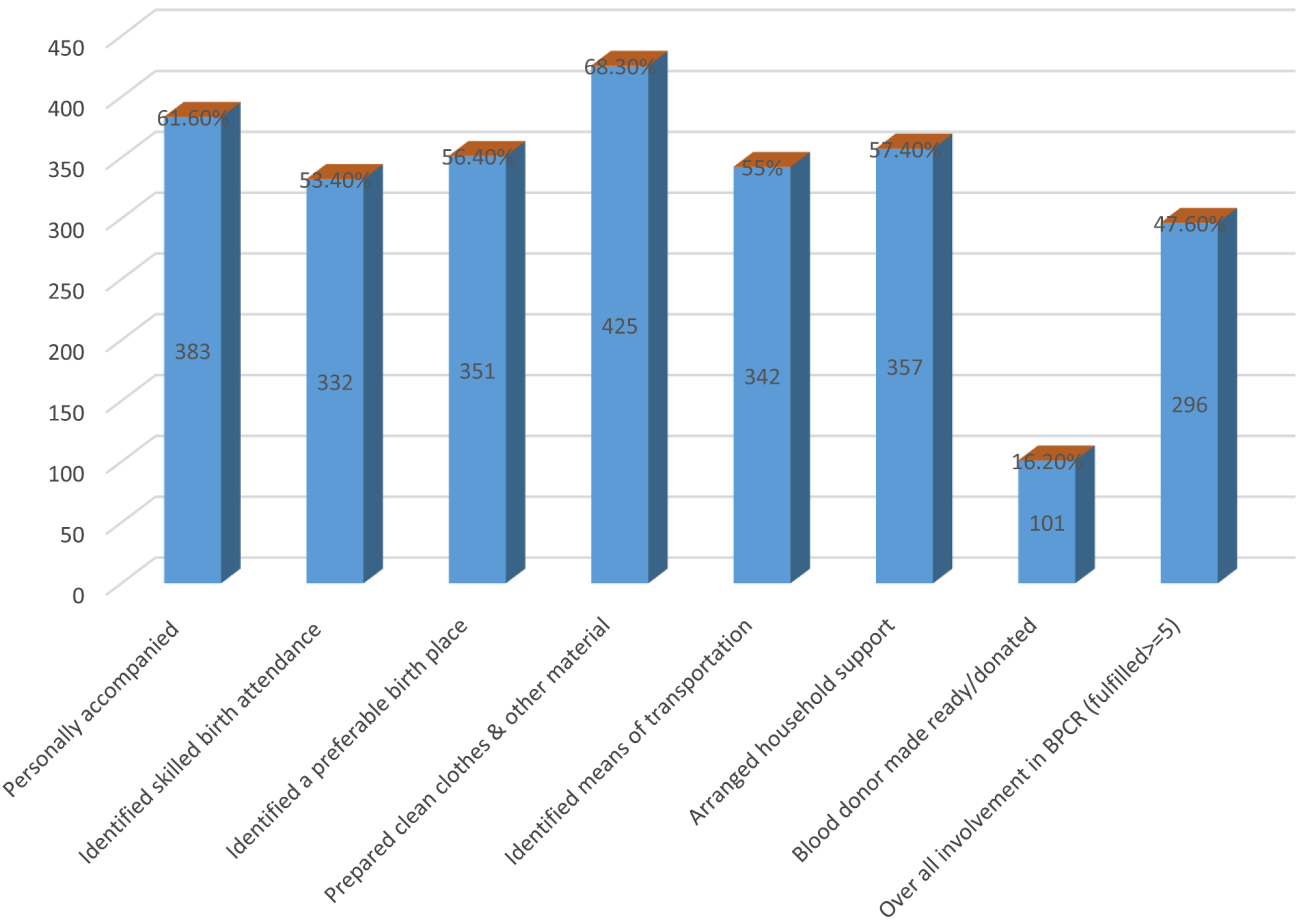
Male partners’ involvement on birth preparedness and complication readiness plan in Dale district, Sidama region, Ethiopia in 2021(*n* = 622)

Overall, male partners’ involvement in birth preparedness and complication readiness plan was estimated at 296 (47.6%). Specifically, the majority (461, or 74.1%) of the study participants saved money for critical time; 332, or 53.4%, of the study participants identified themselves as skilled birth attendants (SBA); 342, or 55.0%, of the study participants identified means of transportation during emergencies; 351, or 56.4%, of the study participants identified their place of delivery; 425, or 68.3%, prepared clean clothes and other material necessary for the mother and newborn baby; and 383, or 61.6%, of the study participants accompanied their wives to a health facility during antenatal care visits. More than half (57.4%) of the study participants arranged household support for temporary family care, and only 101 (16.2%) of the study participants identified as blood donors in case of emergency (See Fig. 1).

### Factors associated with male partners’ participation birth preparedness and complication readiness plan

Table 5 presents the result of logistic regression analysis male partners’ involvement on birth preparedness and complication readiness plan.

**Table 5:** Result of logistic regression analysis of factors associated with male involvement in birth preparedness and complication readiness plan among study participants

Variables Category		Involvement on BPCRP		COR 95% CI	AOR 95% CI	*p*-value
		Yes	No			
Husbands who accompanied wives for antenatal care	No	221	111	1	1	
	Yes	75	215	5.7(4.1, 8.1)*	2.3(1.5, 3.5)**	0.000
Knowledge of male partners about birth preparedness and complication plan	No	203	101	1	1	
	Yes	93	225	4.9(3.5, 6.8)*	3.5(3.1,6.5)**	0.000
Knowledge of male partners on obstetrics danger signs	Poor	127	224	1	1	
	Good	176	75	4.5 (3.2,6.4)*	3.5 (2.2,5.7)**	0.000
Presence of pregnancy previous complication of husbands’ wives	Yes	262	180	6.3(4.1, 9.5)*	5.1(4.3,11.2)**	0.000
	No	34	146	1	1	
Gravidity of husbands’ wives	Primipara	143	41	6.1 (4.1,9.1)*	2.7 (1.6,4.7)**	0.001
	Multipara	160	278	1	1	
Mode of delivery of husband’s wives	C/S	23	9	2.9(1.4, 6.5)*	2.1(1.1,3.8)**	0.027
	SVD	273	317	1	1	

*Key* BPCRP: Birth Preparedness and Complication Readiness Plan, SVD-Spontaneous Vaginal Delivery, C/S-Cesarean Section, OR-Crude Odds Ratio, AOR-Adjusted Odds Ratio, *=P-value < 0.25, **=P value < 0.05

In this study, all independent variables were checked for having an association with the outcome variable in Crude Odds Ratios (COR); however, only 6 variables were associated statistically in multivariate logistic regression analysis model. These include: husbands who accompanied their wives during antenatal care, knowledge of male partners about birth preparedness and complication readiness plan, knowledge of husbands about pregnancy danger signs, husbands whose wives face previous obstetrics complication, spouse’s number of pregnancy, and mode of delivery were variables associated with male partners’ involvement in birth preparedness and complication readiness plan.

Husbands who accompanied their spouses during prenatal care were 2.3 times more chance to participate in birth preparedness and complication readiness plan than their counterparts (AOR = 2.3, 95%CI: 1.5, 3.5). The chance of involvement in birth preparedness and complication readiness plan among husbands with adequate knowledge was 5.1 times more than their counterparts (AOR = 5.1, 95%CI: 4.3, 11.2). Compared with chance of husbands of women who had no previous pregnancy complication, husbands whose wives who face previous complications had 3.5 times more odds of involvement in birth preparedness and complication readiness plan (AOR = 3.5, 95%CI: 2.2, 5.7). Male partners of primi-gravida women were 2.7 times more likely to engage in birth preparedness and complication readiness plan than their counterparts (AOR = 2.7, 95%CI: 1.6, 4.7). Explicitly, male partners whose spouses had delivered their child through caesarean section were 2.1 more likely to be involved in birth preparedness and complication readiness plan than their counter parts (AOR = 2.1, 95%CI: 1.1, 3.8) (See Table 5).

## Discussion

This study highlights that the prevalence of male partners involvement in birth preparedness and complication readiness plans is 47.6% (95%CI: 44.9%, 48.9%) in Dale district, Sidama Ethiopia, in 2021.This finding is higher than the prevalence of male partners’ involvement in birth preparedness and complication readiness plan in low income countries [10], and Kucha Gamo, South Ethiopia [23].This pattern of result might be due to variation in study population, study tool, government strategies and policies, health system capacity, population lifestyle and culture of societies among African countries.

This finding is lower than the findings of studies done in Ethiopia: Wolayta, South Ethiopia (55%) [24], Debre Birhan City, Central Ethiopia (51.4%) [20], Axum (64.7%) [11], and Mekelle, North Ethiopia (62%) [24]. The possible reason may be explained by the difference in residence status among male partners. Most of the study participants in the current study were rural dwellers. Male partners who reside in the countryside had inadequate information due to a lack of technology, infrastructure, and access to health facilities [25, 26]. Variation in culture and lifestyle of both couples in rural populations is influenced by male partners’ involvement in the health decision-making process [27]. The current finding is consistent with the findings of the studies done in rural Uganda (42.9%) [28] and low-income African countries (42.4%) [10]. The results strongly imply overlapping strategies for maternal health services and the commitment of local health facility professionals [22]. This idea is further supported by the finding that partners of women who live in urban areas have better maternal health service utilization than their counterparts [21].In this study, most male participants were involved in preparing clean clothes and other materials (68.3%). This finding is lower than finding of study done in Mekelle (80.1%) [24]. The possible difference might be associated with level of education, community perception towards wives support. However, minorities (18.2%) of male partners were involved in blood donation. This finding is lower than finding of study done in Wolayta Sodo, South Ethiopia (30%) [25]. In this study, vaginal bleeding is commonest known danger sign during pregnancy (66%), labour (67.4%) and post-partum period (69.1%). In my view, the most compelling explanation for the present set of findings is that might be associated with the fact that vaginal bleeding is more common manifestation with immediate outcome and the commonly known danger sign by community health workers so that communicate it for community members. On the contrary, this finding is higher than finding of study conducted in Wolayta Sodo South Ethiopia (6%) [26].

60% (60%) of the study participants know that the placenta was not delivered 30 min after the baby was born during labor and delivery. This result is consistent with the findings of a study done in Ghana [27]. On the other hand, convulsion is the least known danger sign during pregnancy (42%), labor, and delivery (52%). This finding is less than the findings of the study done in Burayu, Oromia region, Central Ethiopia (27.2%) [18]. This might be associated with differences in the level of health information access among communities. There are three key findings of the present research: male partners who accompanied their wives during antenatal care visits were more likely to be involved in birth preparedness and complication readiness plan than their counterparts. This idea is further supported by the findings of the study in Ambo, North Shewa, and Ethiopia [28]. Antenatal care represents a window of opportunity for information, education, and communication with pregnant women so that they will make appropriate choices during pregnancy, especially when they are in danger. However, this opportunity is often missed [25]. Male partners who had good knowledge of birth preparedness and complication readiness plan were more likely to engage with birth preparedness and complication readiness plan than their counterparts. This finding was in line with the findings of studies conducted in Wolayta town and Mekelle town [20, 25]. Taken together, our findings indicate that knowledge is the basic element for behavioral changes [25]. Male partners whose spouses had a history of cesarean sections were more involved in birth preparedness and complication readiness plan than their counterparts. This finding is consistent with the findings of the study done in Uganda [27]. This finding may be explained by the idea that the presence of partners during such complications in health facilities and subsequent comprehensive health information access, fear of future complications, and uncertainty could push male partners to engage in birth preparedness and complication readiness plan extensively [26, 27]. Male partners’ whose spouse faced obstetric complications were more likely to participate in birth preparedness and complication readiness plan than their counterparts. This finding is consistent with previous findings of the study done in Wolayta, South Ethiopia [26]. The possible rationale might be that obstetric complications can have a long-lasting impact on the memories of male partners, which could improve their health-seeking behavior. Hence, male partners might be involved in birth preparedness and complication readiness plan as a mitigation action for future risks. Finally, male partners of primi**-**gravida women had a greater chance to engage in birth preparedness and complication readiness plan than their counterparts. This finding is in agreement with the findings of a study conducted in Uganda [27]. Male partners’ overemphasis and lack of confidence in different circumstances during pregnancy and childbirth might trigger them to be involved in the care and support of their wives.

### Limitation of the study

This study could be suffered from potential problem of recall and social desirability bias. However, authors have mitigated this issue by limiting participation of partners’ of wives who gave birth in the last 12 months only. On the contrary, this study also has a number of strengths of assessing male partners’ participation during birth preparedness and complication readiness plan for the most affected population. Future researchers should focus on the barriers of male partners’ participation on maternal health and male partner’s opinion on birth preparedness and complication readiness plan using qualitative approach.

### Public health implication

These data have some potential intervention implications. For example, policy makers should design strategies and public health policies which increase men participation in maternity health care. Effective community health education programs should be designed by integrating men participation on maternal health.

## Conclusion

Male partners’ involvement on birth preparedness and complication readiness plan was low. Male partners’ knowledge of birth preparedness and complication readiness plan, male partners’ accompaniment of their wives during antenatal care, husbands whose wives had previous caesarean section, the presence of previous obstetrics complications among husbands’ spouses, husbands with primi-para wives, and male partners’ good knowledge about obstetrics danger signs of pregnancy were factors significantly associated with male involvement on birth preparedness and complication readiness plan.

Therefore, advocating of participation of husbands in maternal health should be the key intervention for male involvement on birth preparedness and complication readiness plan. Besides, health education about obstetrics danger sign for male could enhance male partners’ engagement on birth preparedness and complication readiness plan.

## Data Availability

The datasets used and/or analyzed during the current study available from the corresponding author on reasonable request.
